# Antibody secreting B cells and plasma antibody response to rotavirus vaccination in infants from Kolkata India

**DOI:** 10.1016/j.heliyon.2018.e00519

**Published:** 2018-02-01

**Authors:** Anuradha Sinha, Suman Kanungo, Deok Ryun Kim, Byomkesh Manna, Manki Song, Ju Yeon Park, Bisakha Haldar, Prashant Sharma, Aiyel Haque Mallick, Soon Ae Kim, Sudhir Babji, Dipika Sur, Gagandeep Kang, Mohammad Ali, William A Petri Jr., Thomas F Wierzba, Cecil Czerkinsky, Ranjan Kumar Nandy, Ayan Dey

**Affiliations:** a National Institute of Cholera and Enteric Diseases, Kolkata, India; b International Vaccine Institute, Seoul, South Korea; c Department of Microbiology and Immunology, Seoul National University; d Division of Gastrointestinal Sciences, Christian Medical College, Vellore, India; e Johns Hopkins Bloomberg School of Public Health, Baltimore, USA; f The University of Virginia, Charlottesville, VA, USA; g PATH, Washington, DC, USA; h Institut de Pharmacologie Moleculaire & Cellulaire, CNRS-INSERM-University of Nice-Sophia Antipolis, Valbonne, France

**Keywords:** Medicine, Infectious disease, Vaccines, Immunology

## Abstract

**Background:**

Assessing immune response after rotavirus vaccination consists in measuring serum or plasma IgA and IgG antibodies, but these assays provide very little information about the mucosal immune response. Thus the development of assays for detection of mucosal immune response following rotavirus vaccination is essential. We evaluate to assess circulating antibody-secreting cells (ASCs) as a potential means to evaluate mucosal immune responses to rotavirus vaccine.

**Methods:**

372 subjects, aged 6 weeks, were enrolled in the study. All the subjects were assigned to receive two doses of Rotarix^®^ vaccine. Using a micro-modified whole blood-based ELISPOT assay, circulating rotavirus type-specific IgA- and IgG-ASCs, including gut homing β7+ ASCs, were enumerated on week 6 before the first dose of Rotarix vaccination at 7 weeks of age and week 18 after the second vaccination at 17 weeks of age. Plasma samples collected before vaccination, and after two doses of Rotarix^®^ vaccination were tested for plasma rotavirus IgA titers.

**Results:**

Two doses of Rotarix^®^ provided to induce sero-protective titer of ≥ 20 Units in 35% of subjects. Total blood IgA- ASC responses were detected in 26.4% of subjects who were non-responder before vaccination. Among responders, 47% of the subjects also have sero-protective plasma IgA titers.

**Discussion:**

Our results suggest that virus-specific blood gut homing ASCs were detected and provide insight into mucosal immune response after rotavirus vaccination. Further studies are needed to evaluate the duration of such immune responses and to assess the programmatic utility of this whole blood-based mucosal ASC testing for the rotavirus immunization program.

## 1 Introduction

Globally, one out of ten children below 5 years of age dies due to diarrheal diseases, resulting in 800,000 fatalities annually. Most deaths occur in sub-Saharan Africa and South Asia. Among these deaths, rotavirus (RV) is the leading cause of severe gastroenteritis and is responsible for 215,000 deaths per year with most of the deaths occur in developing countries [1]. RV pathogenesis involves RV replication inside enterocytes causing pathological changes in enterocyte membrane inducing malabsorptive or osmotic diarrhea. Mucosal immunity is considered to provide protection from RV entry and replication. Intracellular viral replication can be inhibited by secretory anti VP6-immunoglobulin A (IgA) before transcytosis across the membrane of enterocytes [2].

Among the currently licensed oral RV vaccines, Rotarix^®^ and RotaTeq^®^ are known to have high efficacy against severe RV disease or RV associated hospitalization in high and middle income countries but lower efficacy in developing countries like India, Bangladesh, Malawi, South Africa etc. Oral rotavirus vaccine (RotaTeq^®^) was only 58% effective at preventing severe rotavirus infection in Nicaragua, compared to > 98% in Finland [3]; while the Rotarix^®^ was found to be ∼95% effective in Europe [4], but only 77% in South Africa [5], 43% in Bangladesh [6] and 49% in Malawi [5]. Further, the recently developed Rotavac^®^ has only 53.6% in India with reduced immunogenicity of 40% [7].

Little is known about the mediators of protective immunity and correlates of protection for RV. A strong local intestinal immune response in the form of secretory immunoglobulin-A (sIgA) is necessary for vaccine efficacy against enteric diseases. These responses are measured indirectly by determining serum or plasma IgA levels. Serum or plasma anti-RV antibodies have been used in several RV vaccine trials [5, 8, 9, 10] and accepted as a marker of vaccine immunogenicity and a possible surrogate of protection in the community; however for individuals there is no recognized correlate of protection [11]. Cut-off of ≥ 20 U/ml rotavirus-specific IgA antibody is considered protective for RV infection [12, 13]. Low efficacy of RV vaccine in some of the high risk populations has called into question, whether plasma anti-RV IgA levels are sufficient in assessing immune protection after RV vaccination. Alternative methods for assessing mucosal immunity have been explored including measurement of RV-specific antibodies in mucosal excretions/secretions such as feces, breast milk and saliva samples [14, 15, 16]. To date, none of these methods have gained general acceptance as mucosal correlates (or surrogates) of immune protection against RV.

Our approach is to measure RV immunity by quantification of plasma anti RV IgA titers and circulating antigen-specific antibody-secreting cells (ASCs) expressing mucosal homing receptors [17, 18]. Antigen specific activation of the B cells redirect them from the secondary lymphoid organs to the effector tissues. Since RVs replicate in the enterocytes so the immune response generate in the intestine and the effector functions are carried out in the intestinal mucosa. Thus the measurement of ASCs harboring the intestinal homing receptor α4β7+ after vaccination could provide information on rotavirus infection or vaccination and these data could complement other measures of immunity to predict RV vaccine immunogenecity. These ASCs appear in blood transiently ∼ 7 days after infection or vaccination and can be measured by enzyme linked immunospot (ELISPOT) assay [17]. To better understand this relationship, we integrate RV vaccine intervention within a large cohort study in Kolkata, India [19]. The primary objective of this study was to measure seropositivity (i.e. plasma anti RV IgA titers) in infants from this region after two doses of rotavirus vaccination. The secondary objective was to explore the frequency of RV-specific blood ASCs, including gut-derived β7^+^ ASCs, as an early marker of mucosal response following RV vaccination.

## 2 Material and methods

### 2.1 Study design and methods

The written informed consent from the parent of each infant was obtained. The study was approved by the Indian Council of Medical Research (ICMR), Health Ministry's Screening Committee (HMSC), Government of India; the Institutional Ethical Committee at National Institute of Cholera and Enteric Disease (NICED) and the Institutional Review Boards of International Vaccine Institute (IVI), South Korea; University of Virginia and University of Vermont USA. The study protocol was registered at clinical trial registry of India (CTRI/2012/03/002504) and at clinicaltrials.gov (ClinicalTrials.gov Identifier: NCT01571505).

This study was nested within a clinical trial titled the Performance of Rotavirus and Oral Poliovirus Vaccines in Developing Countries (PROVIDE) study. Total 372 Infants of 6 weeks age were recruited at a children’s hospital in urban slums of Kolkata, India from March 28, 2012, to October 30, 2013. The subjects were followed up for 53–54 week of infant age, and final follow up completed on November 30, 2014. All subjects received diphtheria, pertussis and tetanus (DPT), Hepatitis B, and Oral Polio Vaccine (OPV) as part of routine immunization. Subjects were randomized to receive OPV and Inactivated Polio Vaccine (IPV) at 39 weeks of age. Additionally, all subjects were vaccinated with Rotarix^®^ as a part of the study [19]. Blood specimens (2 ml) were collected by venipuncture at baseline (i.e. week 6), and at week 18 after vaccination with two doses of RV vaccine (Rotarix^®^) given at 10 and 17 weeks of age and samples were tested for plasma anti RV IgA ELISA and B-cell ELISPOT. The reason for selection of 17 week boost was to harmonize with PROVIDE study arm of Bangladesh, where delayed dosing of rotavirus vaccine were done in community with high incidence of rotavirus diarrhea [20]. Further blood collection on week 18 was done to measure rotavirus immunogenicity using the plasma IgA and B-cell ELISPOT assay. In this study we were interested in measuring gut homing antibody secreting cells (ASCs) after rotavirus vaccination using ELISPOT assay. These cells are short lived and can be detected in blood as early as 5 days after vaccination, peaking most often on day 7, and then rapidly decreasing to become undetectable after 2 weeks [17]. Thus, we collected blood samples at week 18, one week after second vaccination dose. Due to ethical restriction on the number of blood draws from infants, we did not draw another blood samples after 4 weeks of second vaccination for repeat measuring plasma IgA response.

### 2.2 ELISA for detection of anti-RV IgA antibodies from blood

Plasma samples were tested for IgA antibody titers to RV using a sandwich ELISA as described earlier [21]. Briefly, 96 well plates were coated with rabbit anti-RV IgG and incubated overnight at 4 °C. The rabbit anti RV IgG was developed in house by CMC Vellore and use at 1:1500 dilution. Next day the plates were washed and to the alternate rows RV strain WC3 cell culture lysate and mock antigen/control (MA104 cell line lysate only) were added after diluting 1:1 in 1% skim milk. Plates were incubated at 37 °C and followed by wash with PBS tween. Standard positive control plasma with known titers (250 units/ml) were diluted from 1/40 to 1/5120 dilution. For test samples, 1/20 to 1/160 dilution were used. Plasma test samples and standard positive control were added to both rows with RV WC3 and MA104 cells lysate. Negative control sample at 1/20 and 1/40 dilution were also added to the plate. Following incubation for 1 hour at 37 °C, plates were washed and anti-human IgA was added to each well. Plates were washed again and antibody labelled with peroxidase were added and incubated at 37 °C for 40 min. The wells in the plate were subsequently developed with *O*-phenylenediamine dihydrochloride (Sigma) in 0.1 M citrate buffer containing 0.01% H_2_O_2_. Reaction was ceased by the addition of 6 N H_2_SO_4_ and the optical density was measured using an ELISA reader (Multiscan, ThermoFisher) at 492 nm. The anti RV IgA antibody titers in units (U)/ml were measured by comparing the optical density values from sample wells with a standard curve based on standard positive control obtained from Christian Medical College (CMC) Vellore, India. Seropositivity was defined as a ≥ 20 U/ml anti RV IgA titer after vaccine administration [12, 13, 21].

### 2.3 ELISPOT for detection of anti-RV ASC from blood

Measurements of blood ASC responses were performed at NICED immune-monitoring laboratory on 2 ml samples of anti-coagulated blood collected at week 6 and one week after first booster dose at week 17. All samples were processed within 4 hours after collection. A micro-modified version of the original ELISPOT assay was used for simultaneous detection of magnetically enriched blood ASCs secreting IgA or IgG to RV (double layered rotavirus particles) [17, 22, 23]. Briefly, 1 ml of EDTA-treated blood was lysed with a red blood cell lysis solution for 5 minutes, washed with PBS-EDTA buffer following centrifugation, and re-suspended to the initial blood volume (1 ml) with PBS-EDTA buffer. Human ASCs were enriched from lysed blood using a mixture (1:1) of magnetic beads coated with monoclonal antibodies to HLA-DR (Mouse anti-human HLA-DR antibody, BD, 1 mg/ml, cat. 555809)and CD19 (Dynabeads^®^ CD19 Pan B), followed by application of a magnetic field [24]. These ASCs are being referred to as “total” ASCs. “Mucosal ASCs” were isolated from 1.0 ml lysed blood samples using magnetic beads coated with monoclonal antibodies to β7+ (Rat Anti-Human- Integrin β7+, antibody, BD, Cat. BV650). After magnetic capture, free and cell-bound beads were washed and re-suspended to the original blood volume in plasma‐free medium prior to being assayed for ASC numbers. These separation procedures routinely yield negative fractions depleted by more than 95% of FACS-detectable HLA-DR^+^, CD19^+^ cells and by more than 90% β7^+^ cells [25], respectively. Depending on the cells retrieval, 2500–5000 cells were used for determining total immunoglobulin spots and 15,000–20,000 cells were used for detecting antigen specific spots. Moreover, negatively sorted cells contain less than 1% ELISPOT detectable immunoglobulin-secreting cells (ISCs).

For antigen-specific ASC enumerations, ELISPOT wells previously coated with purified double layered rotavirus particles [22] and control antigen (i.e. Bovine Serum Albumin, BSA) were prepared. Apart from ASCs, immunoglobulin-secreting cells (ISCs) irrespective of antigen specificity were detected in parallel wells coated with a mixture of affinity-purified goat antibodies to human Ig k and λ light chains [17]. All coated plates were dried and kept in individual sealed aluminum bags with a desiccator prior to being used (within 3 months). After incubation of ASC- and ISC-containing cell suspensions for 3 h at 37 °C in a CO_2_ incubator, wells were extensively washed with PBS-EDTA and PBS-Tween 20. Next, a mixture of appropriately diluted goat antibodies to human IgA and IgG, respectively labeled with alkaline phosphatase and horseradish peroxidase, was added to the wells and incubated for one hour. After washings, zones of solid phase-bound secreted IgA and IgG antibodies were visualized by stepwise incubation with corresponding enzyme chromogen substrates followed by washing with water [17]. After drying, plates were scanned for blue (IgA) and red (IgG) spots with an automated ELISPOT reader.

Total and mucosal ASCs as well as ISCs were enumerated against RV antigens and net ASC and ISC counts were determined after subtracting corresponding non-specific counts detected in control (BSA-coated) wells. Data are expressed as ASC or ISC numbers per milliliter of blood.

### 2.4 Statistical analyses

Numbers of RV-specific IgA-and IgG-producing blood ASCs were determined at week 6 and week 18. ASC responses were further differentiated into total (HLA-DR+/CD19+) and mucosal (β7+) ASCs.

We calculated geometric mean concentration (or spots) and 95% confidence interval of the outcomes. A subject was considered as ASC responder if his/her count had at least 5 spots per 10^3^ ISC^26^. Statistical analyses were performed using SAS 9.3 (SAS Institute, Cary NC).

## 3 Results

**Fig. 1:**
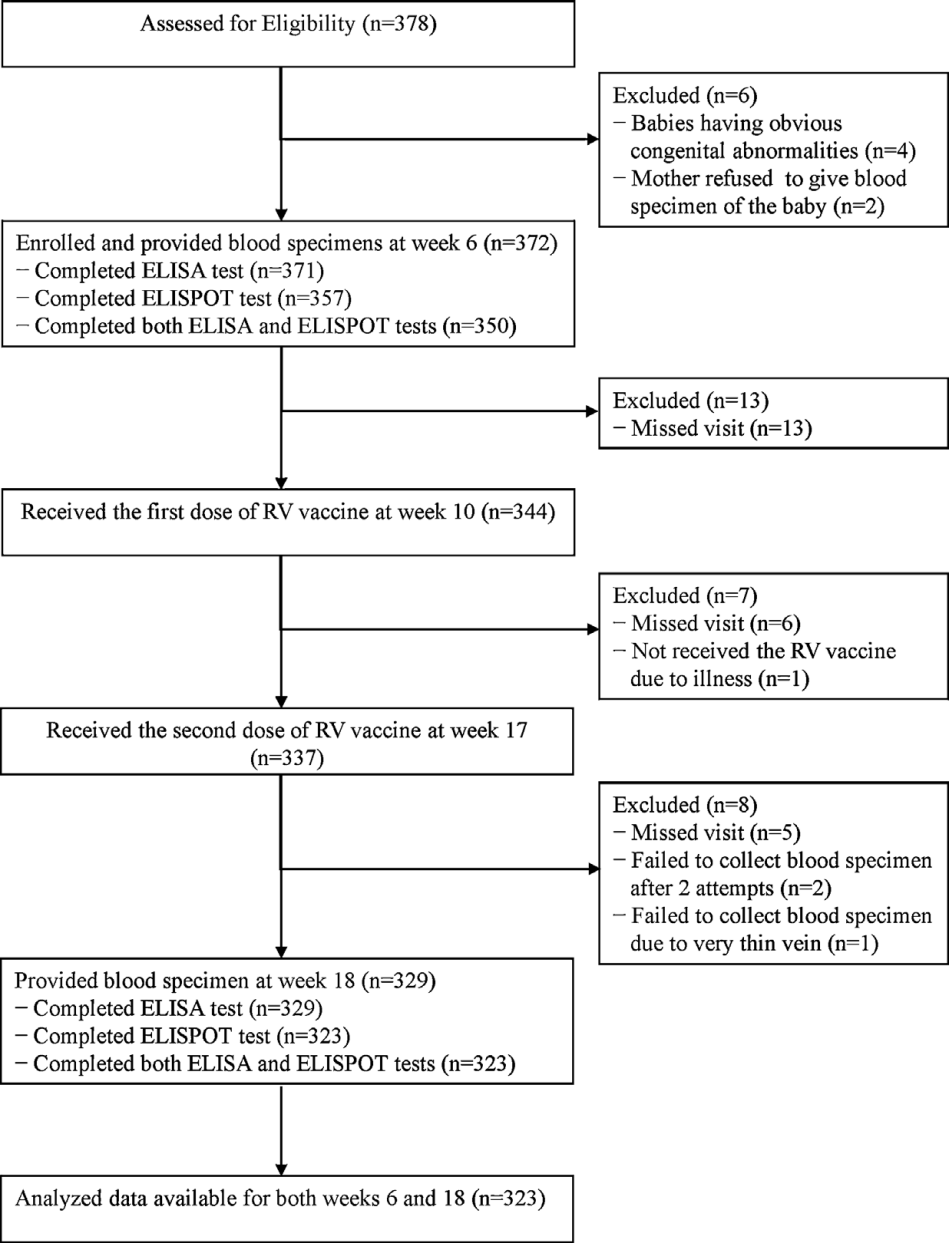
Consort flow diagram of included subjects.

A total 323 subjects (86.8%) had a complete dataset and were included in the analysis out of 372 subjects enrolled in this study. Subjects were excluded if they were lost to follow-up, if we failed to collect blood sample after several attempts or before due to thin vein, or if blood quantity was not sufficient for testing (Fig. 1).

### 3.1 Serum IgA response to rotavirus vaccination

Of the enrolled subjects, 28/323 (9%) were seropositive at the baseline having ≥ 20 U/ ml of anti-RV IgA. Among the seronegative subjects, 88/295 (30%) seroconverted after two doses of Rotarix vaccine. (Table 1).

**Table 1:** Rotavirus-specific plasma IgA antibody titers before and after Rotarix vaccination.

		Week 18 (Post second dose)			
Seropositivity status		Post second dose ≥ 20U/ml		Post second dose < 20U/ml	
Week 6 (Baseline)	No. of infants (%)	No. of infants (%)	GMC^a^(95% CI)	No. of infants (%)	GMC^a^(95% CI)
Baseline ≥ 20U/ml	28 (9%)	25 (89%)	97.31 (60.89, 155.5)	3 (11%)	12.37 (7.92, 19.32)
Baseline < 20/ml	295 (91%)	88 (30%)	57.94 (47.98, 69.97)	207 (70%)	1.58 (1.23, 2.04)

a GMC, Geometric mean concentration in U/ml.

### 3.2 Blood ASC response to rotavirus vaccination

**Fig. 2:**
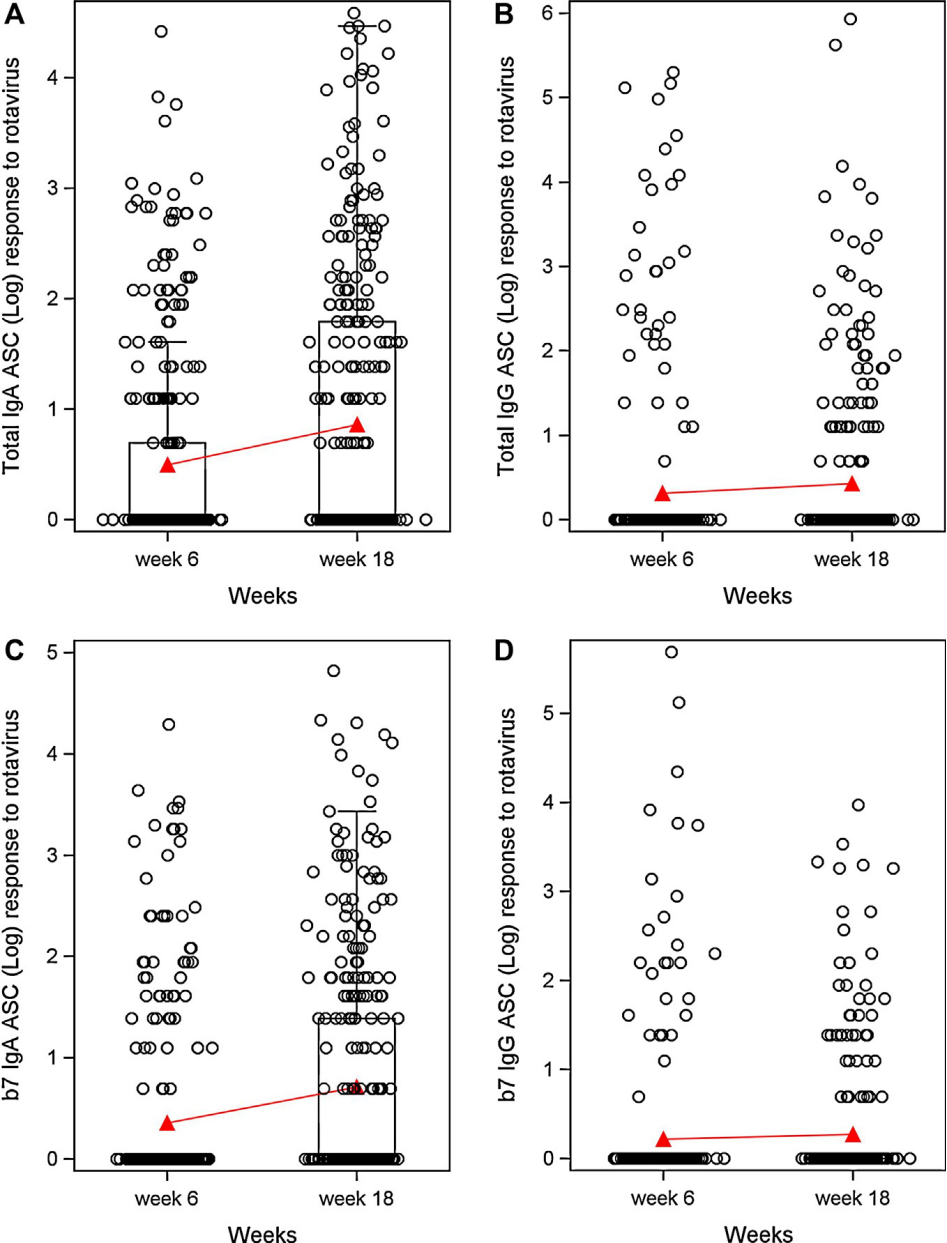
**A**: The figure depicts distribution of ASCs (O) and the geometric mean titres (columns with error bars) of rotavirus specific total (HLADR+/CD19+) IgA, before and after vaccination with Rotarix^®^ [The red line depict the trend in increase in rotavirus specific ASCs after vaccination]. **B**: The figure depicts distribution of ASCs (O) and the geometric mean titres (columns with error bars) of rotavirus specific total (HLADR+/CD19+) IgG, before and after vaccination with Rotarix^®^ [The red line depict the trend in increase in rotavirus specific ASCs after vaccination]. Columns with error bar not visible because of large number of non-responders among the subjects. **C**: The figure depicts distribution of ASCs (O) and the geometric mean titres (columns with error bars) of rotavirus specific gut homing (β7 +) IgA, before and after vaccination with Rotarix^®^ [The red line depict the trend in increase in rotavirus specific ASCs after vaccination,]. **D**: The figure depicts distribution of ASCs (O) and the geometric mean titres (columns with error bars) of rotavirus specific gut homing (β7 +) IgG, before and after vaccination with Rotarix^®^ [The red line depict the trend in increase in rotavirus specific ASCs after vaccination]. Columns with error bar not visible because of large number of non-responders among the subjects.

Before RV vaccination at week six, 46/304 (15%) subjects and 28/304 (9.2%) subjects were found to be responders by RV specific total IgA ASCs and total IgG ASCs, respectively, and 37/304 (12.2%) and 20/304 (6.6%) subjects were found to be responders by RV specific β7+ IgA ASCs and β7+ IgG ASCs, respectively (Tables 2 and 3). Among the nonresponder subjects, 68/258 (26.4%) responded for total IgA ASCs after two doses of Rotarix vaccine with GMSpot of 44.02. Similarly 57/267 (21.3%) get seroconverted for β7+ IgA ASC with GMSpot of 42.84 (Tables 2 and 3, Fig. 2A, B, C and D). We did not observe an increase in total and gut homing IgG ASCs after vaccination.

**Table 2:** Rotavirus specific total IgA ASC (HLADR/CD19) & β7+ IgA ASC responses.

Total IgA (n=304)^c^					
Immune status		Week 18 (Post second dose)			
		Responder^a^		Non-Responder	
Week 6 (Baseline)	No. of infants (%)	No. of infants (%)	GMSpot^b^(95% CI)	No. of infants (%)	GMSpot^b^(95% CI)
Responder^a^	46 (15.1%)	15 (32.6%)	38.01 (23.82,60.64)	31 (67.4%)	1.28 (0.96, 1.69)
Non-Responder	258 (84.9%)	68 (26.4%)	44.02 (35.30,54.90)	190 (73.6%)	1.63 (1.41, 1.88)

**β7+ IgA (n=304)**^c^					
**Immune status**		**Week 18 (Post second dose)**			
		**Responder**^a^		**Non-Responder**	
**Week 6 (Baseline)**	**No. of infants (%)**	**No. of infants (%)**	**GMSpot**^b^**(95% CI)**	**No. of infants (%)**	**GMSpot**^b^**(95% CI)**

Responder^a^	37 (12.2%)	7 (18.9%)	67.94 (30.10,153.3)	30 (81.1%)	1.52 (1.06, 2.19)
Non-Responder	267 (87.8%)	57 (21.3%)	42.84 (33.67,54.51)	210 (78.7%)	1.57 (1.37, 1.79)

a Responder was defined as 5 spots or more per 10^3 HLADR ISC.

b GMSpot, Geometric mean spot.

c Some samples were excluded from the analysis due to non-appearance of spots at HLADR ASC well: 3 samples of IgA and 10 samples of IgG at week 6, and 10 samples of IgA and 23 samples of IgG at week 18.

**Table 3:** Rotavirus specific total IgG ASC (HLADR/CD19) & β7+ IgG ASC responses.

Total IgG (n=304)^c^					
Immune status		Week 18 (Post second dose)			
		Responder^a^		Non-Responder	
Week 6 (Baseline)	No. of infants (%)	No. of infants (%)	GMSpot^b^(95% CI)	No. of infants (%)	GMSpot^b^(95% CI)
Responder^a^	28 (9.2%)	7 (25%)	39.61 (14.34,109.5)	21 (75%)	1.39 (0.86, 2.24)
Non-Responder	276 (90.8%)	26 (9.4%)	27.77 (19.23,40.12)	250 (90.6%)	1.39 (1.25, 1.55)

**β7+ IgG (n=304)**^c^					
**Immune status**		**Week 18 (Post second dose)**			
		**Responder**^a^		**Non-Responder**	
**Week 6 (Baseline)**	**No. of infants (%)**	**No. of infants (%)**	**GMSpot**^b^**(95% CI)**	**No. of infants (%)**	**GMSpot**^b^**(95% CI)**

Responder^a^	20 (6.6%)	6 (30%)	22.89 (13.75,38.12)	14 (70%)	1.64 (0.93, 2.88)
Non-Responder	284 (93.4%)	17 (6.0%)	35.24 (22.87,54.32)	267 (94.0%)	1.26 (1.16, 1.38)

a Responder was defined as 5 spots or more per 10^3 HLADR ISC.

b GMSpot, Geometric mean spot.

c Some samples were excluded from the analysis due to non-appearance of spots at HLADR ASC well: 3 samples of IgA and 10 samples of IgG at week 6, and 10 samples of IgA and 23 samples of IgG at week 18.

Detection of vaccine responders differed by assay. Among the 88 total IgA ASC responders, 41 (47%) subjects had seroprotective plasma titers, while among 76 β7+ IgA responders, 41 (55%) subjects have seroprotective plasma titers. Of the subjects negative for plasma responses, 47 (53%) of 88 were positive by total IgA ASCs and 34 (45%) of 75 were positive by β7+ IgA ASC. We observed seroprotective titers in 67 (30%) total IgA and 67 (28%) β7+ IgA ASC negative responders. Among all 313 subjects, there were 171 (55%) non-responders by plasma and β7+ IgA, and 158 (50%) non-responders by plasma and total IgA. (Table 4).

**Table 4:** Comparison of Rotavirus ELISPOT responders (≥ 5 spots) and plasma IgA responders (≥ 20 U/ml) at week 18.

			ELISA IgA Responders	
ELISPOT Assays			≥ 20U/ml	< 20U/ml
Responders		N	n (%)	n (%)
β7 IgA	≥ 5 spots	75	41 (55%)	34 (45%)
	< 5 spots	238	67 (28%)	171 (72%)
Total IgA	≥ 5 spots	88	41 (47%)	47 (53%)
	< 5 spots	225	67 (30%)	158 (70%)
Total		313	108 [35%]	205 [65%]

## 4 Discussion

While a previous study has explored the induction of gut homing α4β7+ cells following natural rotavirus infection in children [27], we examined the role of antigen-specific ASCs including gut-homing ASCs following RV vaccination in humans. As described elsewhere [28], blood ASCs are plasma blasts, precursors of tissue plasma cells, and the effector component of the humoral immune response [29, 30, 31]. Upon infection or vaccination, these ASCs migrate to lymphoid tissue or specific site, secreting IgA or IgG antibodies and can be detected in the blood for short period of time [32]. The detection of these ASCs in blood provides an early marker of exposure to foreign antigens in peripheral and mucosal tissues [24, 33] and when assessed at an early time point, at around one week after immunization, can assess vaccine responses and subsequent immunological memory [34]. Furthermore, ASC precursor B cells activated at mucosal sites express tissue-specific homing receptors directing their selective migration to specific mucosal tissues [35, 36].

ASCs induced by mucosal immunization mainly express β7+-integrin [35]. Integrin β7+ is associated with α4 (CD49d) unit, expressed on subsets of lymphocytes and in thymus on mucosal sites. It also associates with CD103 (Integrin α IEL) expressed on T cells adjacent to mucosal epithelium and intraepithelial lymphocytes. The integrin α4β7+ is an important receptor that mediates trafficking of lymphocytes to intestinal lymphoid tissues and is important for the homing of plasmablasts to the gut [36]. In mice lacking β7+, plasma cell numbers in the intestinal lamina propria are reduced many-fold [37]. The induction of gut homing integrin β7+ cell secreting IgA to rotavirus may play a crucial role since these β7+ cells produce polymeric IgA, the precursor of secretory IgA antibodies.

Similar to natural infection [27], β7^+^ IgA- ASC responses and total IgA- ASC responses were observed after two doses of vaccine, suggests mucosal homing of β7^+^ ASCs from and to the gut. More specifically, the number of infants with β7^+^ IgA- ASC doubled and total IgA-ASCs more than double from baseline to one week after the second dose. Still, the proportion of responders for both plasma antibody and ASC responses following two vaccine doses was low. These results are similar to the results of other investigations exploring responses to this vaccine, but consistent with earlier studies conducted in south Indian infants [8] and in north Indian infants [9]. The low response rate may reflect the effects of tropical enteropathy [38] a disease of the gut that dampens immune responses to antigens and possible due to constant fecal-oral contamination, waning of gut immunity due to pre-exposure presence of maternal antibodies and presence of other enteric pathogens, interference with other vaccines, and host characteristics (e.g., genetics, level of nourishment, breastfeeding), [39, 40].- Further in our study, plasma IgA antibody response was measured at one week post second dose, there was limited time to induce antibody production on all subjects."

In previous RV studies, seroconversion rate showed around 35% after dose 1 and increased to around 50–60% after dose 2, in South Africa [41]. The best time to measure anti-RV IgA antibody response is at least 4 weeks after second dose vaccination. Similar observation was also from Rotarix^®^ vaccine trial in India, where 58.3% subjects have ≥ 20 anti-RV IgA titer 4 weeks of immunization [13]. However, as described earlier, due to ethical restriction in our study on number of blood draws, plasma samples were tested for IgA antibody response only one week after vaccination.

There was evidence of pre exposure before vaccination as observed from the high ASC response at week 6 in some of the subjects which is also reflected by anti RV plasma IgA in our study. Extending the analysis to IgG class, even fewer responders in both gut homing and circulating IgG ASCs were observed. The low IgG ASC response following RV vaccination can be explained as a part of the compartmentalization of the mucosal immune response in the respiratory and the digestive tract. In our study, vast majority of these ASCs have a gut homing characteristic and secrete IgA. This is supported by earlier observation that despite having similarities in adhesion molecule expression between IgG and IgA ASC, the gut homing cells induced following mucosal priming pre-dominantly are IgA secreting cells [24].

Worldwide RV infection occurs in childhood especially under five years of age but occurs much earlier in the developing world [42, 43]. This fact is understandable through the previous reports which detected ∼40% RV infection in hospitalized neonates [44]. Thus the baseline seropositivity found in our study may be related to pre-exposure and is in concordance with other studies evaluating immunogenicity of Rotavirus vaccines. But interestingly in the present study in Kolkata area the baseline seropositivity is much lower than found in other studies in other parts of India [8]. Based on the fact that high baseline seropositivity is indicative of ongoing exposure, measuring plasma IgA levels may not be fully informative in terms of differentiating immune-protection following RV vaccination and natural infection. Thus arises the need for complementary assays which can specifically measure mucosal and cellular markers of immune responses after RV vaccination, independent of serological antibody levels detected.

In the present study the stimulation of RV-specific blood ASCs, and especially β7+ ASCs, following rotavirus vaccination was demonstrated. Comparison of ASC responses with anti RV plasma IgA titers ended into four groups of ASC positive/plasma IgA positive, ASC negative/plasma IgA negative, ASC positive/plasma IgA negative and ASC negative/plasma IgA positive, suggesting that the ELISPOT assay may be complementary but not duplicative of plasma IgA measurement. Data from the present study suggests, ability of blood β7+ IgA and/or IgG ASCs to measure mucosal immune protection against rotavirus was moderate. This could be due, in part to the time frame (7 days post vaccination) selected for blood collection which may have been suboptimal as these ASCs may reaches peak earlier or later than 7 days post vaccination. Further exploring a range of time points of blood collection could help improving the predictive ability of this marker. Furthermore, important study limitations must be mentioned. As this was an exploratory study, we haven’t included any non-vaccinated or placebo arm for comparison. Moreover, we compare the mucosal ASC response to plasma IgA titers which is not a true correlate of mucosal immune protection. Diarrheal surveillance following vaccination and detection of rotavirus in stool of vaccinated vs non-vaccinated could help prediction of true mucosal protection following vaccination. Also measurement of mucosal antibody secretions and gut T-cell response need to be considered. Further exploring a range of time points for blood collection for ELISPOT analysis and plasma IgA response could help validate such an association as some children may respond earlier than 7 days and considering plasma IgA can be optimally induce after 4 weeks of vaccination.

The primary advantage of utilizing the ELISPOT assay is that it requires a small volume of blood (1–2 ml) for assessing ASC response; this is particularly helpful in infants and children where there is a restriction of withdrawing only small blood volume [45]. The current approaches to determining circulating ASCs require a relatively large volume of blood (3–5 mL) [17], and a flow cytometry-based technique is comparably more expensive and requires more technical skills with advance laboratory setup. Our current method overcomes these limitations by enrichment and selection of ASCs from a small volume of 1 mL of blood, followed by an ELISPOT analyses.

In summary, this study explored virus-specific blood ASCs, including β7+ ASCs, as a marker for RV vaccine-induced mucosal gut homing response, which can be detected in a small blood volume. This study also indicates that blood ASCs provide an early marker of the mucosal and systemic immunogenicity of these vaccines. The results suggest that oral vaccine studies might include determination of gut homing ASCs to measure immune responses following vaccination, in addition to measurement of systemic antibody titers for better understanding of the type of immune response induce. Furthermore, ASC response demonstrates compartmentalization of immune system [17, 26] and may play important role in developing strategies for vaccine delivery and alternate route of immunization.

## Declarations

### Author contribution statement

Anuradha Sinha: Performed the experiments; Wrote the paper.

Deok Ryun Kim, Byomkesh Manna, Ju Yeon Park, Aiyel Haque Mallick: Analyzed and interpreted the data.

Manki Song: Analyzed and interpreted the data; Wrote the paper.

Bisakha Haldar, Prashant Sharma: Performed the experiments.

Soon Ae Kim, Sudhir Babji: Performed the experiments; Contributed reagents, materials, analysis tools or data.

Dipika Sur: Conceived and designed the experiments; Contributed reagents, materials, analysis tools or data.

Gagandeep Kang: Contributed reagents, materials, analysis tools or data.

Mohammad Ali: Conceived and designed the experiments; Analyzed and interpreted the data;

Suman Kanungo, William A Petri Jr., Cecil Czerkinsky: Conceived and designed the experiments.

Thomas F Wierzba: Conceived and designed the experiments; Wrote the paper.

Ranjan Kumar Nandy, Ayan Dey: Conceived and designed the experiments; Performed the experiments; Analyzed and interpreted the data; Contributed reagents, materials, analysis tools or data; Wrote the paper.

### Funding statement

This work was supported by University of Virginia, through grant from Bill and Melinda Gates Foundation (Grant No. OPP1017093). Additional support to International Vaccine Institute is provided by the governments of the Republic of Korea and Sweden. Also support to AD through National Research foundation of Korea (http://www.nrf.re.kr), grant number 2013K1AZA1058633 is gratefully acknowledged.

### Competing interest statement

The authors declare no conflict of interest.

### Additional information

The clinical trial described in this paper was registered at the clinical trial registry of India under the registration number CTRI/2012/03/002504 and at clinicaltrials.gov under the registration number NCT01571505.
